# Indicaxanthin from *Opuntia ficus-indica* Fruit Ameliorates Glucose Dysmetabolism and Counteracts Insulin Resistance in High-Fat-Diet-Fed Mice

**DOI:** 10.3390/antiox11010080

**Published:** 2021-12-29

**Authors:** Simona Terzo, Alessandro Attanzio, Pasquale Calvi, Flavia Mulè, Luisa Tesoriere, Mario Allegra, Antonella Amato

**Affiliations:** 1 Dipartimento di Scienze e Tecnologie Biologiche, Chimiche e Farmaceutiche, Università degli Studi di Palermo, Via Archirafi, 28, 90123 Palermo, Italy; simona.terzo01@unipa.it (S.T.); alessandro.attanzio@unipa.it (A.A.); pasquale.calvi@unipa.it (P.C.); flavia.mule@unipa.it (F.M.); luisa.tesoriere@unipa.it (L.T.); antonella.amato@unipa.it (A.A.); 2 Dipartimento di Biomedicina, Neuroscienze e Diagnostica Avanzata, Università degli Studi di Palermo, Via del Vespro, 129, 90127 Palermo, Italy

**Keywords:** indicaxanthin, *Opuntia ficus-indica*, phytochemicals, insulin resistance, obesity, inflammation, oxidative stress, dysmetabolism

## Abstract

Obesity-related dysmetabolic conditions are amongst the most common causes of death globally. Indicaxanthin, a bioavailable betalain pigment from *Opuntia ficus-indica* fruit, has been demonstrated to modulate redox-dependent signalling pathways, exerting significant anti-oxidative and anti-inflammatory effects in vitro and in vivo. In light of the strict interconnections between inflammation, oxidative stress and insulin resistance (IR), a nutritionally relevant dose of indicaxanthin has been evaluated in a high-fat diet (HFD) model of obesity-related IR. To this end, biochemical and histological analysis, oxidative stress and inflammation evaluations in liver and adipose tissue were carried out. Our results showed that indicaxanthin treatment significantly reduced body weight, daily food intake and visceral fat mass. Moreover, indicaxanthin administration induced remarkable, beneficial effects on HFD-induced glucose dysmetabolism, reducing fasting glycaemia and insulinaemia, improving glucose and insulin tolerance and restoring the HOMA index to physiological values. These effects were associated with a reduction in hepatic and adipose tissue oxidative stress and inflammation. A decrease in RONS, malondialdehyde and NO levels, in TNF-α, CCL-2 and F4-80 gene expression, in p65, p-JNK, COX-2 and i-NOS protein levels, in crown-like structures and hepatic inflammatory foci was, indeed, observed. The current findings encourage further clinical studies to confirm the effectiveness of indicaxanthin to prevent and treat obesity-related dysmetabolic conditions.

## 1. Introduction

Obesity is a major, global health problem, affecting approximately 500 million adults and 40 million children worldwide [1]. Prospective studies highlight that obesity increases the risk of several pathological conditions, such as type-2 diabetes (DM), hypertension and coronary heart disease, being responsible for almost 3 million deaths every year [2]. Obesity stems from a chronic imbalance between energy intake and expenditure and involves the accumulation of excessive body fat within adipose tissue. Due to its spectacular complexity, as both a nutrient sink and endocrine organ, adipose tissue is a key district where metabolic regulations and immunological responses are highly integrated and the proper function of each depends on the other. Along these lines, an obesity-induced disruption of this delicate equilibrium results in the development of inflammatory-dependent, dysmetabolic conditions, including insulin resistance (IR), DM and non-alcoholic fatty liver disease (NAFLD) [3].

IR is the reduced ability of an organism to mount a normal and coordinated glucose-lowering response via tissue-autonomous and crosstalk-dependent mechanisms [4]. From a mechanistic perspective, IR originates from a chronic caloric imbalance that generates adipose tissue hypertrophy and hyperplasia [5,6,7]. In response to hypertrophic signals, adipocytes have the innate capacity to secrete pro-inflammatory adipokines and hormones, establishing a state of chronic, systemic, low-grade inflammation [3,8,9]. The increased release of these mediators, then, stimulates the recruitment, retention and activation of M1 macrophages within the white adipose tissue itself. Here, the activation of pro-inflammatory signalling pathways, involving both c-Jun NH2-terminal kinase (JNK) and NF-κB, leads to the elaboration of paracrine mediator system, including tumour necrosis factor-α (TNF-α), interleukin-1β (IL-1β) and IL-6. As a result, the release of these pro-inflammatory cytokines into the bloodstream impairs insulin signalling in the hypertrophic adipocytes, thus increasing adipocyte lipolysis. The chronically increased efflux of non-esterified fatty acids (NEFA) favours their ectopic deposition (lipotoxicity) in the liver and skeletal muscle [4,10,11,12]. On the other hand, excessive storage of the intrahepatic fat that characterises hepatic steatosis is regarded as an obesity-associated liver pathology strictly linked with IR [12,13]. The IR state, caused by pro-inflammatory (JNK- and NF-κB-dependent), oxidative and lipotoxic mediators, is initially accompanied by a compensatory increase in pancreatic β cell insulin secretion for the maintenance of euglycemia [13,14]. If compensatory insulin secretion fails, β cells collapse, becoming unable to produce sufficient insulin, and a transition from IR to DM occurs.

In recent times, *Opuntia ficus-indica* cladodes have been exploited for nutraceutical and health-promoting purposes since they exert a plethora of beneficial effects on both lipid and glucose dysmetabolism [15,16,17,18,19,20]. Conversely, much less attention has been paid to the fruit, hallmarked by a phytochemical fingerprint different from the cladode one. *Opuntia ficus-indica* fruits are, indeed, enriched in the betalains pigments, exclusively present within Caryophyllales and in some genera of higher fungi, wherein they replace anthocyanins. Amongst the betalains of *Opuntia ficus-indica* fruit, indicaxanthin has been investigated over the last 20 years for its biochemical, pharmacological and nutraceutical properties [21]. This phytochemical, an adduct of betalamic acid with proline, is highly bioavailable in humans [22]. Indeed, the ingestion of a single serving of the yellow cultivar of *Opuntia ficus-indica* fruit generates, in humans, an indicaxanthin plasma peak concentration of 7 µM after 2 h. Relevantly, thanks to its reducing and amphipathic properties, indicaxanthin was shown to interfere with cellular, redox-dependent signal transduction pathways in several experimental models of inflammatory-related, oxidative stress-dependent pathological conditions [21,23]. Along these lines, significant reducing, anti-oxidative, anti-inflammatory, anti-proliferative, anti-tumoral, spasmolytic and neuromodulatory and neuroprotective [24] effects of indicaxanthin have been reported both in vitro and in vivo [21]. Interestingly, NF-κB has been proposed as one of the crucial molecular targets that indicaxanthin can interact with, to exert its anti-inflammatory effects [25,26,27,28,29].

In light of the strict interconnections between obesity, oxidative stress, inflammation, IR and hepatic steatosis, and taking into account the redox-modulating and anti-inflammatory properties of indicaxanthin, we here investigated the potential beneficial effects of the phytochemical in an in vivo animal model of diet-induced obesity. To this end, a nutritionally relevant dose of indicaxanthin, extracted from the yellow cultivar of *Opuntia ficus-indica* fruits, was evaluated, in mice fed a high-fat diet (HFD), which progressively develops a pathology similar to human metabolic syndrome, including obesity, hyperglycaemia, IR and hepatic steatosis [30,31]. In particular, indicaxanthin’s impact was evaluated on glucose and lipid dysmetabolism, as well as on oxidative stress and inflammation.

## 2. Materials and Methods

Unless otherwise specified, all reagents and chemicals were purchased from Merck (Milan, Italy) and of the highest purity grade available.

### 2.1. Extraction and Purification of Indicaxanthin

Indicaxanthin was extracted from *Opuntia ficus-indica* fruits (yellow cultivar, San Cono, Sicily, Italy) as detailed in the Italian Patent Application No. 102021000015167 filed on 10.06.2021. Briefly, the fruits were peeled and finely chopped, the pulp was separated from the seeds and weighed, and 100 g pulp samples were homogenised and centrifuged at 3000× *g* for 10 min. The supernatant was recovered while the pellet was extracted with 100 mL of distilled water and centrifuged as above reported. The combined supernatants were subjected to cryodesiccation, and the phytochemical, in the resulting aqueous extract, was separated by size exclusion chromatography on a Sephadex G-25. Fractions containing the pigment were subjected to cryodesiccation, followed by solid-phase extraction (SPE) on J.T. Baker, Bakerbond SPE C18 columns (VWR, Milan, Italy). The eluate was subjected to rotary evaporation to remove methanol and the residue dissolved in phosphate-buffered saline (PBS). Indicaxanthin concentration was assessed by spectrophotometric revelation at 482 nm with an extinction coefficient of 48 mM^−1^ cm^−1^ [27]. All samples were portioned and stored at −80 °C until further use.

### 2.2. Animals

Four-week-old male C57BL/6 J (B6) mice (*n* = 24) purchased from Envigo (San Pietro Al Natisone Udine, Italy) were housed under standard conditions of light (12 h light:12 h darkness cycle) and temperature (22–24 °C), with free access to water and food. Mice were allowed to acclimate for 1 week prior to the implementation of the special diets. The animals were randomly assigned to a diet group: either to a standard diet (STD) (code 4RF25, Mucedola, Milan, Italy) or to a high-fat diet (HFD) with 60% of energy derived from fat (code PF4051/D, Mucedola). After 10 weeks on their respective diet, the HFD group was further randomly subdivided into further sub-groups: one group fed an HFD and the other one fed an HFD and receiving indicaxanthin orally at a calculated dose of 0.4 mg/kg twice a day for 4 weeks (HFD+IND group). During the 14 weeks of the experiment, changes in body weight and food intake, determined by measuring the difference between the pre-weighed chow and the weight of chow at intervals of 24 h, were measured weekly and results from the different groups of animals were compared. At the end of the experimental protocol, biochemical analyses were performed; then, the animals were weighed and sacrificed by cervical dislocation. Adipose tissue and liver were removed and weighted. One part of each tissue, fixed in 4% neutral formalin solution, was used for the histological analysis and another part was stored at −80 °C for the biomolecular analyses.

### 2.3. Biochemical Analyses

Plasma triglyceride and cholesterol concentrations were measured by using Biochemistry Analyzer MultiCare (Biochemical Systems International-Srl, Arezzo, Italy). Plasma glucose levels were measured using a commercial glucometer (GlucoMen LX meter, Menarini, Italy) in blood collected from the tail vein. Plasma insulin was quantified by a mouse ELISA kit (Alpco diagnostics, Salem, NH, USA). Intraperitoneal glucose tolerance test (IPGTT) and insulin tolerance test (ITT) were carried out in mice fasted overnight. For IPGTT, the animals were injected intraperitoneally (i.p.) with glucose (2 g/kg body weight) in 0.9% saline. For ITT, mice were given an i.p. injection of insulin (0.5 U/kg body weight) (Insuman Rapid, Sanofi Aventis, Italy) in 0.9% saline. Blood glucose was measured at different time intervals (0, 15, 30, 60, 120 min from the administrations). The Homeostasis Model Assessment of basal Insulin Resistance (HOMA-IR) was calculated as the product of fasting insulin (ng/mL) and fasting glucose (mg/dL) divided by the constant 22.5.

### 2.4. Quantification of Hepatic Lipids

Total liver lipids were determined as previously described [29]. Briefly, ~1 g of tissue hepatic sample was homogenised in 25 mL ice-cold chloroform:methanol (2:1) solution for 1 min. The homogenate was centrifuged at 3000× *g* for 10 min to collect the supernatant. For removal of polar lipids, the solvent was washed with 25% of total volume NaCl solution (0.9%), vortexed vigorously for 30 s and centrifuged at 2000× *g* for 5 min in order to separate the two phases. The upper phase was discarded and the lower phase containing the fat was collected and evaporated in a rotary evaporator under vacuum. The weight difference between the starting empty tube and the tube containing the dried lipids was the lipid amount.

### 2.5. Liver and Adipose Tissue Histology and Immunohistochemistry

Liver and visceral white adipose tissues (WAT) were isolated and fixed in 4% formaldehyde solution. The samples were then embedded in paraffin and sliced at a thickness of 5 μm. Liver and WAT morphology was evaluated by staining with haematoxylin/eosin. Under the light microscope, 5 liver fields were chosen at random and were analysed for inflammation assessment, counting the infiltrating cell aggregates in the liver parenchyma at a magnification of 20×. The adipocyte number per fat deposit was determined at a magnification of 20×. Oil Red O staining was performed in frozen liver sections to detect the presence of fat. For Oil Red O staining, livers were snap-frozen, embedded at an optimum cutting temperature and sectioned on a cryostat microtome. Adipocyte size was determined in µm^2^ using image analysis software (Visilog 6, Courtaboeuf, France), with each cell being individually identified and measured. Images of the liver and WAT sections were acquired using a light microscope (Leica DMLB, Meyer instruments, Houston, Texas) furnished with a DS-Fi1 camera (Nikon, Florence, Italy) and were analysed at 10× and 20× magnification. For the immunohistochemistry, adipose tissue sections were deparaffinised in xylene and endogenous peroxidase activity was depleted with 3% hydrogen peroxide for 30 min at room temperature. Sections were then washed in PBS and incubated overnight with primary antibody Mac-2 (1:2800, Cedarlane, ON, Canada CL8942AP). Then, the sections were washed three times with PBS and incubated with the biotinylated secondary antibody (Anti-Mouse IgG/Rabbit IgG) (1:400, Vector Laborato-ries, BA-4001) for 30 min at room temperature. ABC Reagent included in the Elite ABC kit (Vector Laboratories, Burlingame, CA, USA) and diaminobenzidine (Sigma, Milano, Italia) were used according to provided protocols. The presence and numbers of crown-like structures (CLS) were recorded and expressed as number of CLS/10,000 adipocytes.

### 2.6. Reverse Transcription Polymerase Chain Reaction (RT-PCR)

Total RNA from liver and visceral adipose tissue was extracted using the RNeasy Plus Mini Kit (Qiagen, Valencia, CA, USA), following the manufacturer’s instructions. The adipose tissue RNA was first isolated by the Trizol method and then applied to Rneasy columns. cDNA was prepared by reverse transcription of 2 ng of total RNA using the High-Capacity cDNA Reverse Transcription Kit (Applied Biosystems, Waltham, MA, USA). The primers used in PCR analysis are listed in Table 1. The amplification cycles included denaturation for 5 min at 94 °C, denaturation for 45 s at 95 °C, annealing for 45 s at 52 °C and elongation for 45 s at 72 °C. After 40 cycles, the PCR products were separated on a 1.8% agarose gel and visualised by ultraviolet (UV) light using E-Gel GelCapture (Thermo Fisher Scientific, Monza, Italy). The signal intensity of the products was analysed using E-Gel GelQuant Express Analysis Software (Thermo Fisher Scientific, Monza, Italy) and normalised to its respective β-actin signal intensity to obtain the expression levels of the gene targets.

### 2.7. Tissue Homogenates

Liver and adipose tissue were washed in ice-cold 0.9% NaCl and weighted. A 10% (*w*/*v*) homogenate was prepared in ice-cold 40 mM Tris-HCl by using a micro homogeniser [28,32].

### 2.8. Malondialdehyde (MDA) Assay

Evaluation of MDA levels in liver and adipose tissue homogenates was performed according to Ohkawa et al. [33]. Briefly, the reaction mixture contained 0.2 mL of whole homogenate, 0.2 mL of 8.1% sodium dodecyl sulphate (SDS), 1.5 mL of acetic acid solution adjusted at pH 3.5 with NaOH and 1.5 mL of 1% thiobarbituric acid (TBA) aqueous solution. The mixture was finally made up to 4.0 mL with distilled water and heated at 95 °C for 60 min. After cooling with tap water, 1.0 mL of distilled water and 5.0 mL of a n-butanol/pyridine solution (15/1, *v*/*v*) were added, and the mixture was shaken vigorously. After centrifugation at 4000 rpm for 10 min, the absorbance of the organic layer was measured at 532 nm. MDA levels were expressed as nmol MDA/g tissue, using 1,1,3,3, tetramethoxypropane as an external standard.

### 2.9. RONS Assay

RONS levels were detected in liver and adipose tissue homogenates using 2′,7′-dichlorodihydrofluorescein diacetate (H_2_DCF-DA), as previously reported [34]. Briefly, the whole homogenate was centrifuged at 3500 rpm for 10 min at 4 °C and 100 µL of the supernatant was mixed with 5 µL of H_2_DCF-DA (final concentration 10 µM). The mixture was incubated for 30 min at 37 °C, protected from light and the fluorescence intensity was detected at 490 nm (excitation) and 540 nm (emission) by using a plate reader [35].

### 2.10. Nitrite Assay

Nitrogen levels in liver and adipose tissue homogenates were determined using Griess reagent [36].

### 2.11. Western Blot Analysis

To determine the protein levels of insulin receptor β (INSR β), cyclo-oxygenase-2 (COX-2) and inducible nitric oxide synthase (iNOS), liver and adipose tissue samples were homogenised on ice-cold buffer containing 50 mM Tris-HCl (pH 7.4), 150 mM NaCl, 1 mM EDTA, 1% Triton X-100, 24 mM sodium deoxycholate, 0.01% SDS, 10 mM sodium pyrophosphate, 100 mM sodium fluoride, 10 mM sodium orthovanadate, 1.5 μM aprotinin, 1 mM phenylmethanesulfonylfluoride (PMSF) and 2.1 μM leupeptin. The homogenates were centrifuged at 12,000× *g* at 4 °C for 30 min and the supernatants were used for protein determination [37]. Sample buffer (62.5 mM Tris-HCl, 10% glycerol, 2% SDS, 33.2 mM dithiothreitol (DTT) and 0.01% bromophenol blue; pH 6.8) was added to the supernatants. Samples containing 50 μg protein were subjected to SDS-PAGE electrophoresis on 12% acrylamide gels and were then electroblotted onto nitrocellulose membranes. Membranes were blocked for 2 h in 5% (*w*/*v*) skimmed dry milk and subsequently incubated in the presence of the corresponding primary antibodies (Santa Cruz, Milan, Italy, 1:1000 dilution, Table 2) overnight at 4 °C. After incubation for 90 min at room temperature in the presence of secondary, HRP-conjugated antibodies (Dako, Milan, Italy, 1:10,000 dilution), proteins were visualised utilising an enhanced chemiluminescent substrate (1.1 mM luminol sodium salt, 2.0 mM 4-iodophenylboronic acid, 5.3 mM hydrogen peroxide and 0.1 M Tris–HCl, pH 8.6). Chemiluminescent bands were evaluated with a C-Digit Blot Scanner (LI-COR, Lincoln, NE, USA) and band intensities were analysed using LI-COR Image Studio 4.0. 

To determine the protein levels of either cytosolic p-JNK or nuclear p65 subunit, corresponding fractions were prepared according to Seubwai et al. [38]. Briefly, liver and adipose tissue samples were homogenised in hypotonic buffer (10 mM HEPES KOH at pH 7.9, 1.5 mM MgCl_2_, 10 mM KCl, 1 mM EDTA, 1% NP-40, 0.5 mM DTT, 1 mM PMSF and 10 μg/mL aprotinin). After centrifugation at 2600× *g* for 3 min at 4 °C, the supernatant containing the cytosolic fraction was collected. The pellet was used as the nuclear fraction, lysed with nuclear lysis buffer (20 mM HEPES KOH at pH 7.9, 10% glycerol, 420 mM NaCl, 1.5 mM MgCl_2_, 0.2 mM EDTA, 0.5 mM DTT, 1 mM PMSF and 10 μg/mL aprotinin) and incubated on ice for 30 min. The nuclear fraction was obtained by centrifugation at 21,000× *g* for 10 min at 4 °C. Samples of the nuclear and cytosolic fractions containing 50 μg protein were used for analyses of p65 and p-JNK levels as above described. All results were expressed as mean ± SD of the densitometric band analysis obtained from 3 replicates per group. All results were normalised to β-actin. For each biomarker monitored by Western blot, a representative lane was selected to compose the figures.

### 2.12. Statistical Analysis

The results are reported as mean ± SEM. Statistical analysis was performed by ANOVA, followed by Bonferroni post hoc test using Prism 6.0, GraphPad (San Diego, CA, USA). Results with a *p* value < 0.05 were considered statistically significant.

## 3. Results

### 3.1. Impact of Indicaxanthin Treatment on Body Weight

**Figure 1:**
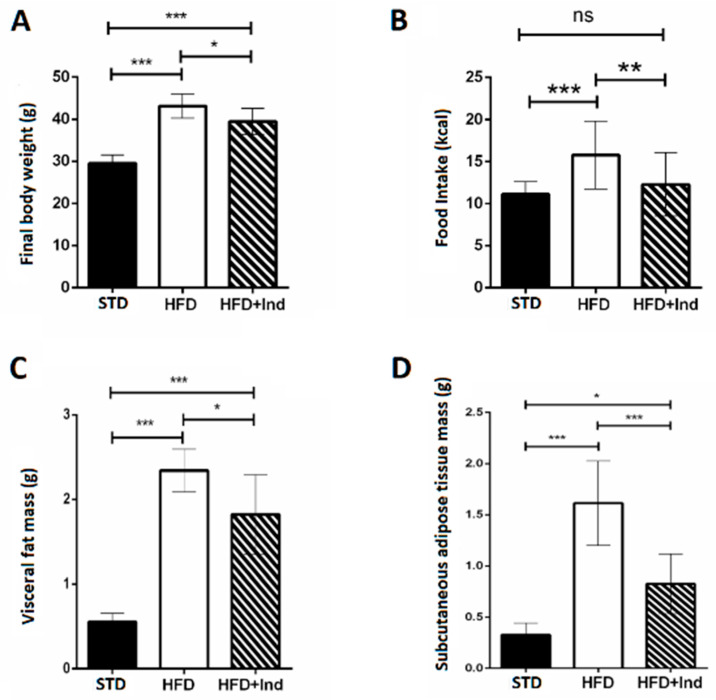
Indicaxanthin treatment reduces body weight gain in HFD mice. Final body weight (**A**), daily food intake (**B**), weight of visceral fat (**C**) and weight of subcutaneous fat (**D**) in the different animal groups. Data are mean values ± SEM of 8 animals/group. ns: *p* > 0.05; * *p* ≤ 0.05; ** *p* ≤ 0.01; *** *p* ≤ 0.001 (ANOVA associated with Bonferroni’s correction).

During the period of study, all mice gained weight. The final body weight reached by HFD mice, the daily food intake and the visceral and subcutaneous mass were significantly higher than those of STD mice. Interestingly, the weight gain, the daily food intake and the visceral and subcutaneous fat mass of indicaxanthin-treated HFD mice were significantly lower than those of HFD animals (Figure 1A–D).

### 3.2. Impact of Indicaxanthin Treatment on Adipocyte Morphology

**Figure 2:**
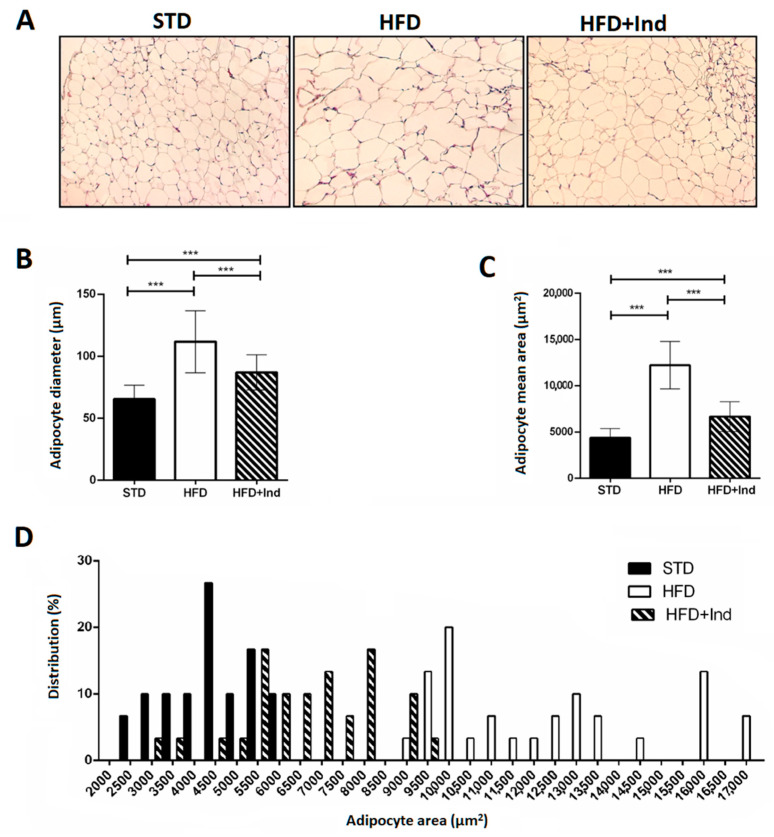
Indicaxanthin treatment reduces visceral adipose tissue hypertrophy in HFD mice. Histological cross-sections of visceral adipose tissue (**A**), adipocyte diameter (**B**), area (**C**) and size distribution (%) (**D**) in the different animal groups. Results are shown as means ± SEM of 8 animals/group. *** *p* ≤ 0.001. (ANOVA associated with Bonferroni’s correction).

Adipocyte area and adipocyte size distribution (%) were analysed in visceral adipose tissue. Adipocyte diameter and area in HFD mice were significantly higher than those in STD mice; however, the degree of increase was significantly reduced in indicaxathin-treated mice, suggesting that indicaxanthin decreases the adipose tissue hypertrophy (Figure 2A–C). The analysis of frequency distribution confirmed this result, revealing that adipocyte sizes in visceral adipose tissue from STD and HFD mice were shifted towards smaller adipocytes after indicaxanthin treatment and thus the proportion of large adipocytes was reduced (Figure 2D).

### 3.3. Impact of Indicaxanthin Treatment on Glucose Dysmetabolism

**Figure 3:**
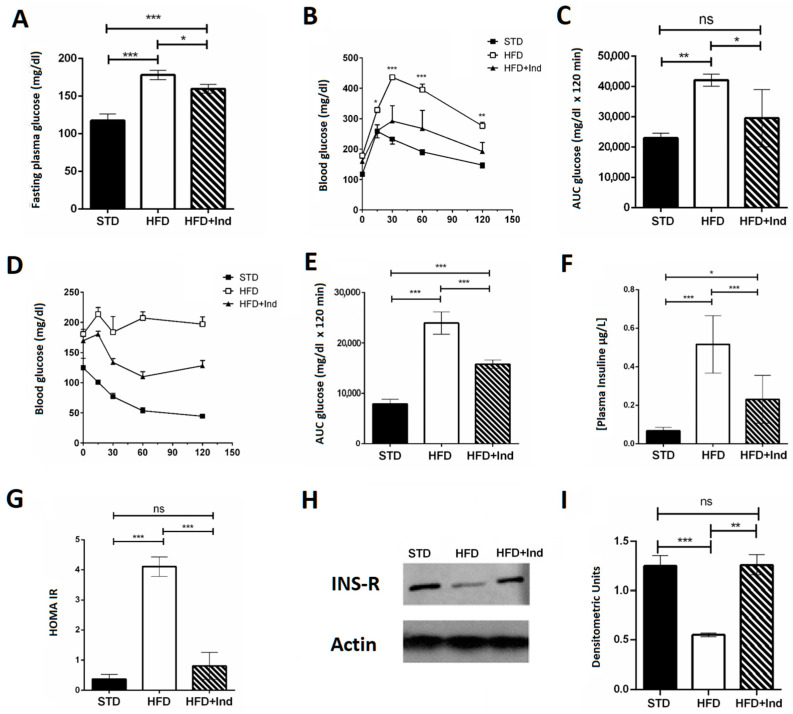
Indicaxanthin treatment improves glucose dysmetabolism in HFD mice. Fasting glycaemia (**A**), glucose tolerance test (GTT) (**B**), area under the curve (AUC) for GTT (**C**), insulin tolerance test (ITT) (**D**), area under the curve for ITT (**E**), plasma insulin levels (**F**), HOMA index (**G**), protein expression levels of insulin receptor and actin in visceral adipose tissue (**H**) and densitometric quantification (**I**). Results are shown as means ± SEM of 8 animals/group. ns: *p* > 0.05; * *p* ≤ 0.05; ** *p* ≤ 0.01; *** *p* ≤ 0.001 (ANOVA associated with Bonferroni’s correction).

Indicaxanthin treatment induced beneficial effects on glucose dysmetabolism. In fact, the indicaxanthin-treated HFD mice showed fasting glycaemia values that were significantly lower than those of HFD mice (Figure 3A). Moreover, they showed improved glycaemic control, as indicated by the reduction in blood glucose levels during the i.p. glucose tolerance test (Figure 3B,C), higher insulin sensitivity as suggested by the insulin tolerance test (Figure 3D,E) and lower plasma insulin concentration (Figure 3F) in comparison with untreated HFD mice. Interestingly, the HOMA index, measured to quantify insulin resistance, was significantly higher in HFD mice than in the STD- or HFD+Ind animal groups (Figure 3G), suggesting that indicaxanthin treatment was able to prevent the insulin resistance induced by HFD consumption. This observation was strengthened by the molecular analysis. Indeed, we found reduced expression of the insulin receptor in the visceral adipose tissue of HFD mice in comparison with STD mice. However, the insulin receptor expression in HFD+Ind mice was significantly higher than that in HFD mice and similar to that in STD animals (Figure 3H,I), confirming that insulin resistance was prevented by indicaxanthin treatment.

### 3.4. Impact of Indicaxanthin Treatment on Lipid Disorders

**Figure 4:**
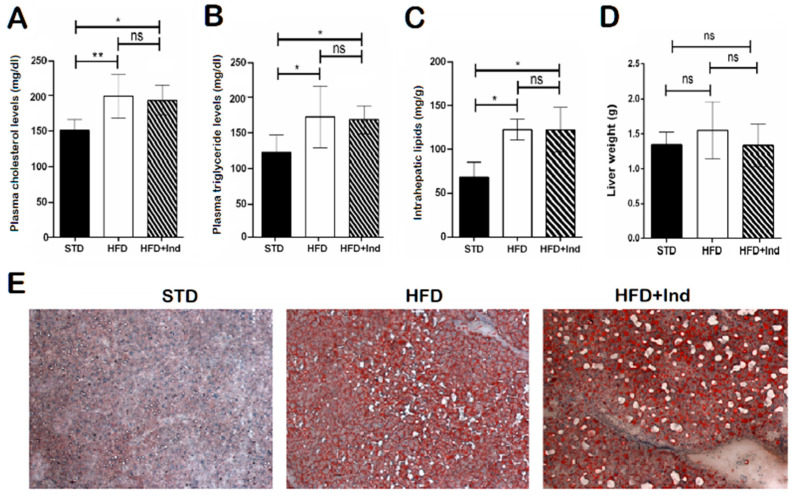
Impact of indicaxanthin treatment on lipid disorders in HFD mice. Plasma cholesterol levels (**A**), plasma triglyceride levels (**B**), intrahepatic lipid levels (**C**), liver weight (**D**), Oil Red O staining of liver sections (**E**) in the different animal groups. Results are shown as means ± SEM of 8 animals/group. ns: *p* > 0.05; * *p* ≤ 0.05; ** *p* ≤ 0.01 (ANOVA associated with Bonferroni’s correction).

As previously shown [32], HFD ingestion for the experimental period caused an increase in the plasma triglyceride and cholesterol levels, at intrahepatic lipid concentrations, without a significant difference in the liver weight (Figure 4A–C). In addition, histological analysis of liver sections stained with haematoxylin and eosin or Oil Red O revealed marked micro- and macrovesicular steatosis in comparison with STD mice (Figure 4D,E). However, indicaxanthin treatment failed to prevent the changes associated with HFD consumption (Figure 4A–E).

### 3.5. Indicaxanthin’s Impact on HFD-Induced Oxidative Stress in Liver and Adipose Tissue

Plenty of evidence clearly demonstrates that the HFD regimen significantly increases hepatic and adipose tissue oxidative stress, contributing to the development of IR and glucose dysmetabolism [39]. To evaluate whether indicaxanthin treatment could affect the HFD-induced oxidative stress, we next assessed the hepatic and adipose tissue levels of MDA, RONS and NO.

**Figure 5:**
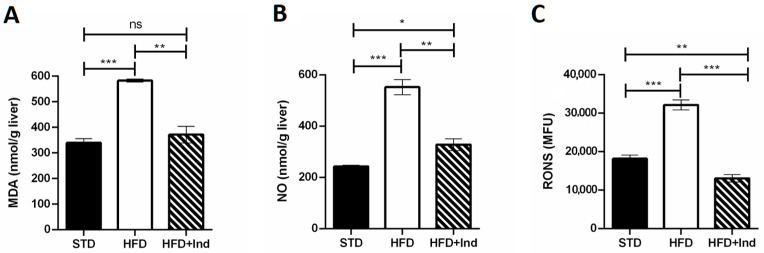

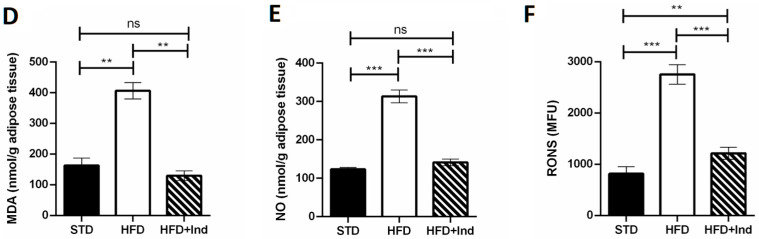
Indicaxanthin treatment prevents oxidative stress in HFD-fed mice. Levels of hepatic (**A**–**C**) and adipose tissue (**D**–**F**) MDA, NO and RONS, respectively. Results are shown as means ± SEM of 8 animals/group. ns: *p* > 0.05; * *p* ≤ 0.05; ** *p* ≤ 0.01; *** *p* ≤ 0.001 (ANOVA associated with Bonferroni’s correction).

As shown in Figure 5, when compared to the STD group, the HFD mice showed a significant increase in all the above-mentioned oxidative stress markers in both liver and adipose tissue (*p* ≤ 0.05, Figure 5A–F). Conversely, indicaxanthin significantly prevented the HFD-induced oxidative stress, in the same tissues, as evidenced by the decreased levels of all the parameters evaluated, in comparison with the HFD group (*p* ≤ 0.05, Figure 5A–F). Notably, treatment with the phytochemical restored hepatic and adipose tissue MDA as well as adipose tissue NO levels to control values (STD group) (Figure 5A,D,E). Moreover, hepatic RONS values were reduced by indicaxanthin treatment even below control values (Figure 5C, *p* < 0.01).

### 3.6. Impact of Indicaxanthin Treatment on HFD-Induced Inflammation in Liver and Adipose Tissue

Chronic subclinical inflammation is a mechanistic link between obesity and IR, leading to alteration of insulin signalling in specific, key, metabolic districts such as the liver and adipose tissue [40]. Along these lines, we next evaluated the impact of indicaxanthin treatment on the HFD-induced inflammation. To this end, adipose tissue CLS density, hepatic inflammatory foci, hepatic and adipose tissue TNF-α, CCL-2 and F4-80 gene expression as well as iNOS, COX-2, pJNK and p65 protein levels were, then, assessed. 

**Figure 6:**
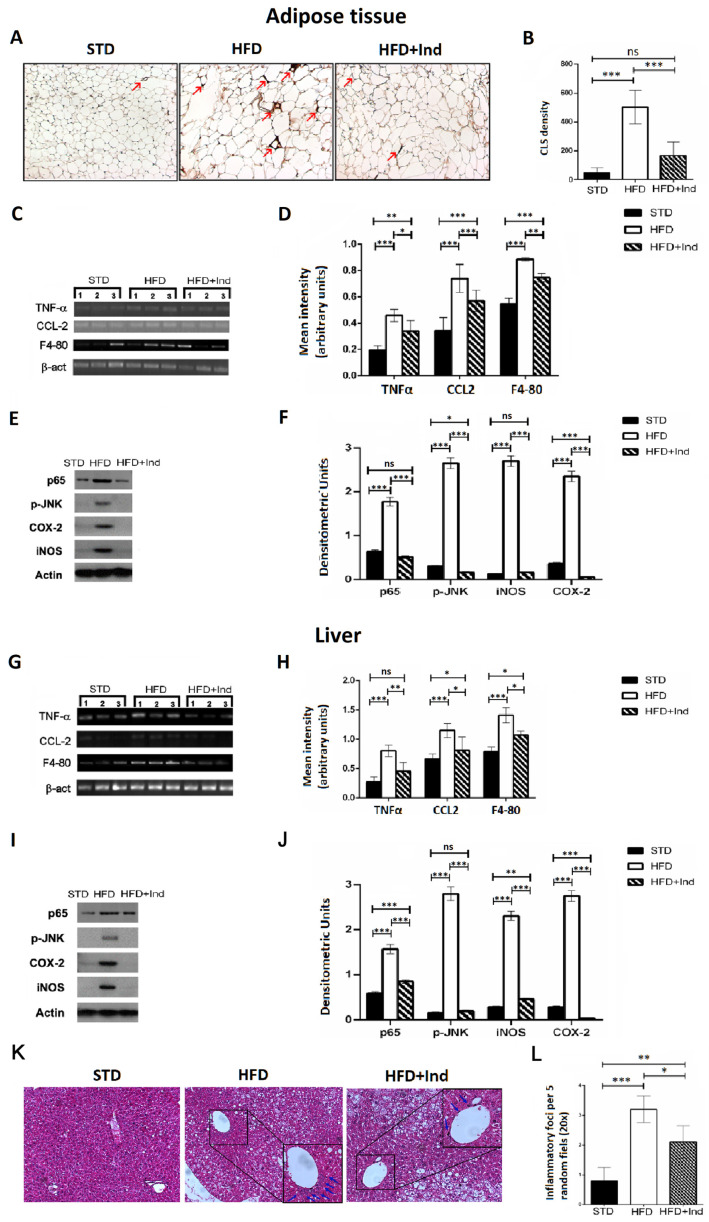
Indicaxanthin treatment prevents inflammation in visceral adipose tissue and liver of HFD-fed mice. Immunohistochemistry analysis in epididymal visceral adipose tissue (VAT) for MAC-2 positive macrophages forming CLS (arrows) (magnification 10×) (**A**); density of MAC-2 positive CLS in VAT (**B**); mRNA expression of TNF-α, F4-80, and CCL-2 and β-actin by PCR in VAT (**C**) and liver (**G**); densitometric analysis of PCR results in VAT (**D**) and liver (**H**); representative Western blot bands of adipose tissue (**E**) and hepatic (**I**) p65, pJNK, iNOS, COX-2 and β-actin protein expression; densitometric analysis of adipose tissue (**F**) and hepatic (**J**) p65, pJNK, iNOS and COX-2 protein levels normalised for β-actin levels; liver histology of examined by H&E staining (**K**). Arrows indicate the points of inflammatory foci (magnification 10×). Quantification of inflammatory foci per 5 random fields under 20× magnification (**L**). Results are shown as means ± SEM of 8 animals/group. ns: *p* > 0.05; * *p* ≤ 0.05; ** *p* ≤ 0.01; *** *p* ≤ 0.001 (ANOVA associated with Bonferroni’s correction).

As shown in Figure 6, when compared to the STD group, the HFD mice showed a significant increase in all the above-mentioned inflammatory parameters, both in the liver and adipose tissue (*p* ≤ 0.05, Figure 6A–L). On the other hand, when compared to the HFD group, the HFD+IND mice showed a significant reduction in macrophage infiltration in visceral adipose tissue, as evidenced by a decrease in CLS density (*p* < 0.001, Figure 6A,B). Accordingly, treatment with the phytochemical significantly decreased cytokine production and other markers of macrophage infiltration, as evidenced by a reduction in TNF-α, CCl-2 and F4-80 gene expression levels in both tissues (Figure 6C,D,G,H). Coherently with the reduced macrophage infiltration, HFD-induced iNOS overexpression was significantly reduced in the liver (*p* < 0.01) and restored to control values in adipose tissue, while COX-2 overexpression was diminished below control values in both tissues (Figure 6E,F,I–L). Notably, indicaxanthin treatment was also able to inhibit HFD-induced NF-κB activation as nuclear levels of p65 were significantly reduced in the liver (*p* < 0.001) and restored to control values in the adipose tissue of the HFD+IND group (Figure 6E,F,I–L). Relevantly, in the same group, these effects were paralleled by a JNK inhibition as the treatment with the pigment reduced adipose tissue p-JNK levels below control values (*p* < 0.05) and restored the hepatic ones to control levels (Figure 6E,F,I–L). Finally, a reduction in the hepatic inflammatory foci number was also induced by indicaxanthin treatment (Figure 6K,L).

## 4. Discussion

This work falls within the remit of intense research on phytochemical-based therapies for the treatment of obesity-associated disorders. Along these lines, we here demonstrate that indicaxanthin, extracted from the yellow cultivar of *Opuntia ficus-indica* fruit, orally administered at a nutritionally relevant amount, prevents and improves obesity-related glucose dysmetabolism and IR in an animal model of metabolic syndrome. Inhibition of HFD-induced inflammation, oxidative stress and NF-κB/JNK activation emerge as key mechanisms underlying the indicaxanthin-mediated benefits.

Consumption of HFD by mice mimics the consequences of Western-style diets in humans in terms of gain weight and obesity [41]. We here observed that mice fed the HFD and treated with indicaxanthin gained less weight than those fed the HFD alone. In line with these results, treatment with the phytochemical prevented WAT fat accumulation as compared to the untreated HFD-fed mice. Indicaxanthin-mediated anti-obesogenic effects are particularly relevant given the role of WAT in the development of systemic adverse effects through the release of adipokines, growth factors and pro-inflammatory mediators [40,42,43].

Several studies have suggested the use of *Opuntia* cladodes as a dietary supplement to prevent obesity, thanks to its fibre content and/or to the presence of phenolic compounds such as quercetin, isorhamnetin or kaempferol [17,18,19]. Relevantly, the current results represent the first experimental evidence for an anti-adiposity effect exerted by a phytochemical of the *Opuntia* fruit. Further experiments are needed to clarify the mechanisms responsible for the anti-obesogenic effects, by verifying the eventual involvement of the hormones controlling food intake.

It is well-known that an increase in adipocyte size induces a functional cellular remodelling that stimulates the secretion of adipokines associated with the development of obesity-associated comorbidities [44,45,46]. Interestingly, our results showed a reduction in the visceral WAT hypertrophia in the indicaxanthin-treated mice that could be linked to the reduction in the inflammatory state observed in the same animal.

On the other hand, the anti-obesogenic effects exerted by the treatment with the yellow betalain were not paralleled by a significant reduction in plasma triglyceride or cholesterol concentrations. Accordingly, no differences were found in the total liver lipid levels between untreated HFD and indicaxanthin-treated HFD mice, ruling out any potential impact of the yellow phytochemical on lipid dysmetabolism. The present evidence on indicaxanthin somehow differs from that in which plant-based whole food and phytochemicals such as polyphenols, alkaloids, flavonoids and saponins exert significant, positive effects on lipid dysmetabolism in vitro or in vivo [32,47,48,49]. Notably, our results suggest a specific effect of indicaxanthin on glucose metabolism.

In our experimental conditions, obesity was associated with the development of hepatic steatosis, as judged by histological analysis in the liver. In fact, consistently with previous studies [50], HFD mice showed microvesicular and macrovesicular steatosis, with large fat droplets in the hepatocytes that displaced the nucleus peripherally and higher lipid accumulation in comparison with STD mice. Coherently with the inability to improve the plasma lipid profile, indicaxanthin treatment also failed to alleviate the structural damage and lipid deposits in the liver caused by HFD feeding. These results further confirm that this phytochemical is not responsible for the benefits on lipid dysmetabolism reported for *Opuntia* fruits both in humans and animal models [51,52,53].

As previously reported, chronic HFD consumption by C57BL/6J mice leads to a profound alteration of glucose metabolism, evident from the increase in fasting plasma glucose and insulin levels, and from the impairment of both glucose and insulin tolerance [41]. A remarkable finding of the present study is that indicaxanthin treatment had a strong impact on glucose dysmetabolism and markedly improved glucose homeostasis. Indeed, we here demonstrate for the first time that treatment with the phytochemical resulted in a significant reduction in fasting glycaemia and insulinaemia and in an improvement in both glucose tolerance and insulin sensitivity. Accordingly, the HOMA-IR index, the most trusted parameter to evaluate the degree of IR, was significantly decreased to control levels. Consistently, the insulin receptor expression was increased by the administration of indicaxanthin, suggesting that IR development was prevented. In terms of biochemical mechanisms, disruption of glucose homeostasis can be envisaged as the result of a self-feeding cycle between systemic, chronic inflammation and oxidative stress, initiated by excess nutrient consumption. Along these lines, one potential explanation for the beneficial effects of indicaxanthin supplementation on insulin sensitivity may lie in its anti-inflammatory properties [25,26,28,54,55].

In obesity-related conditions, low-grade, systemic chronic inflammation is established through the sustained recruitment and infiltration of macrophages in metabolic active tissues [42]. Interestingly, our results clearly show that indicaxanthin treatment reduced liver and adipose tissue macrophage infiltration, decreasing hepatic inflammatory foci, restoring VAT CLS to control levels and reducing F4-80 and CCL2 mRNA expression in both tissues. 

Relevantly, not only did indicaxanthin treatment inhibit the infiltration of macrophages, but it also impaired their activation and the consequent spreading of the inflammatory response in the liver and adipose tissue. Here, levels of HFD-induced, pro-inflammatory mediators, such as TNF-α mRNA and iNOS and COX-2 proteins, were, indeed, significantly reduced by the phytochemical treatment. In this regard, indicaxanthin-induced effects on TNF-α tissue levels are particularly relevant. This cytokine, indeed, plays a key role in the impairment of insulin signalling pathways, reducing insulin-receptor substrate-1 (IRS-1) activation and blocking of GLUT4 translocation [56,57]. Collectively, these data suggest that indicaxanthin treatment has an anti-inflammatory effect in both hepatic and adipose tissues, targeting macrophage infiltration and activation. This hypothesis is consistent with previous evidence from our research group demonstrating that the phytochemical exerts significant anti-inflammatory effects in vivo at the same dose employed in the present study [28] and significantly counteracts macrophage activation in vitro [29].

Upregulation of the cytokines, chemokines and pro-inflammatory enzymes underlying macrophage infiltration and activation is a coordinated process under the control of NF-κB- and JNK-dependent signalling pathways [40,43,58]. Plenty of evidence, moreover, presents NF-κB and JNK as a molecular bridge between inflammation and glucose dysmetabolism. Their activation, indeed, impairs IRS-1 activity, leading to the downregulation of the insulin cascade [4,11,59]. Our results demonstrate that indicaxanthin treatment effectively inhibited HFD-induced NF-κB and JNK activation both in the liver and adipose tissue. Their inhibition could be, therefore, crucial to explain the beneficial effects exerted by the pigment against the HFD-induced macrophage infiltration, activation and IR development. The ability of indicaxanthin to inhibit NF-κB signalling in this HFD model is consistent with our previous observations in other inflammatory-related models, where the yellow betalain was shown to counteract inflammation and tumour progression via NF-κB inhibition, both in vivo and in vitro [26,27,28,29,54,55].

An overwhelming amount of evidence states that the NF-κB and JNK activation process is under the control of endocellular redox modifications [60]. Along these lines, the capacity of indicaxanthin treatment to suppress HFD-triggered NF-κB/JNK activation could be, in part, due to its previously reported ability to inhibit NADPH oxidase and reduce RONS generation [54,55]. In line with this hypothesis, our current results clearly show how indicaxanthin treatment significantly counteracts HFD-induced RONS generation both in the liver and adipose tissue. It has been shown that an HFD-induced RONS increase can enhance MDA levels in the liver and adipose tissue [61]. This reactive aldehyde has been shown to irreversibly form adducts with macromolecules, modifying cell function and contributing to IR development [62,63]. Coherently with the reduction in RONS, the phytochemical treatment also reduced the HFD-induced increase in hepatic and adipose tissue lipid peroxidation, restoring MDA levels to control values. These results appear of interest as they confirm, for the first time in an in vivo context, the already demonstrated ability of indicaxanthin to counteract lipid peroxidation in vitro or ex vivo [22,64]. 

Identifying active components of fruits and vegetables that provide protection against the adverse effects of consuming Western-style diets can have a major impact on human health. Moreover, understanding the mechanisms by which these components act, modifying cell functions, is crucial to define public recommendations in terms of diets and potential supplementation. This work has demonstrated the capacity of indicaxanthin to mitigate the development of obesity and to significantly ameliorate the glucose dysmetabolism and IR promoted by the chronic consumption of an HFD in mice. Our results suggest that the disruption of the HFD-induced cycle of inflammation, oxidative stress and NF-κB/JNK activation can be central in the capacity of indicaxanthin to mitigate HFD-induced glucose dysmetabolism and IR. The current findings encourage further clinical studies to confirm the effectiveness of indicaxanthin supplementation against the adverse health consequences of chronic caloric overload and excessive fat consumption. Moreover, in light of the key role played by IR in neurodegenerative diseases [65] and taking into account the ability of indicaxanthin to cross the BBB [66] and differentially distribute within the central nervous system [67], the present findings motivate further investigations to explore whether the phytochemical can ameliorate central glucose metabolism, thus preventing IR-mediated neurodegeneration.

## 5. Conclusions

As a whole, our results suggest that indicaxanthin, at a nutritionally relevant dose, modulates the expression of crucial genes and proteins involved in the oxidative stress-dependent inflammatory reaction underlying the obesity-related IR. Further studies are necessary to clarify the potential of this nutraceutical as an additive to prevent and treat obesity-related IR in humans and to consider indicaxanthin as a novel, potential, therapeutic agent for obesity-related disorders.

## 6. Patents

Indicaxanthin was isolated from *Opuntia ficus-indica* fruits (yellow cultivar) as detailed in the Italian Patent Application No. 102021000015167 filed on 10.06.2021.

## Figures and Tables

**Table 1:** Oligonucleotide sequence of primers for RT-PCR.

Gene	Forward Primer	Reverse Primer	Size (bp)
TNF-α	5′-AGCCCACGTCGTAGCAAACCA-3′	5′-GCAGGGGCTCTTGACGGCAG-3′	260
F4-80	5′-GCCACGGGGCTATGGGATGC-3′	5′-TCCCGTACCTGACGGTTGAGCA-3′	360
CCL-2	5′-TCTGTGCTGACCCCAAGAAGG-3′	5′-TGGTTGTGGAAAAGGTAGTGGAT-3′	273
β-actin	5′-GGATCCCCGCCCTAGGCACCAGGGT-3′	5′-GGAATTCGGCTGGGGTGTTGAAGGTCTCAAA-3′	289

**Table 2:** Primary antibodies employed in Western blot analysis.

Protein	Catalogue Number	Clone	Size (kDa)
COX-2	sc-376861	H-3	72/70
iNOS	sc-7271	C-11	130
p-JNK	sc-6254	G-7	54/46
p65	sc-8008	F-6	65
INSR β	sc-57342	CT-3	95
β-actin	sc-47778	C4	43

## Data Availability

All of the data is contained within the article.
